# (±)-2′-Phenyl­cyclo­hexa­nespiro-4′-(aze­pano[1,2-*b*]isoxazolidine)

**DOI:** 10.1107/S1600536808021867

**Published:** 2008-07-19

**Authors:** Daryl Crimmins, Ka Wai Choi, Peter D. W. Boyd, Margaret A. Brimble

**Affiliations:** a Department of Chemistry, The University of Auckland, Private Bag 92019, Auckland, New Zealand

## Abstract

In the crystal structure of the racemic title isoxazolidine, C_19_H_27_NO, the relative stereochemistry between the phenyl group and the bridgehead H atom is shown to be *syn*. There are two mol­ecules in the asymmetric unit, one of which is the 7R*,13R* enanti­omer, and one of which is the 7S*,13S* enanti­omer. These enanti­omers adopt different orientations of the phenyl ring with respect to the isoxazolidine ring, with C—C—C—C torsion angles of 63.6 (4) and 86.8 (4)°, respectively. In both enanti­omers, the six-membered ring adopts a chair conformation, while the seven-membered ring adopts a twist-chair conformation.

## Related literature

For related literature regarding the synthesis towards the spiro­imine unit of the spiro­lides, see: Brimble & Trzoss (2004 ▶); Brimble *et al.* (2005 ▶); O’Connor *et al.* (2008 ▶). For the crystal structure of the related 7,6-spiro­lactam unit, see: Guéret *et al.* (2008 ▶). For isolation of spiro­lides from natural resources, see: Hu *et al.* (2001 ▶); MacKinnon *et al.* (2006 ▶); Ciminiello *et al.* (2007 ▶).
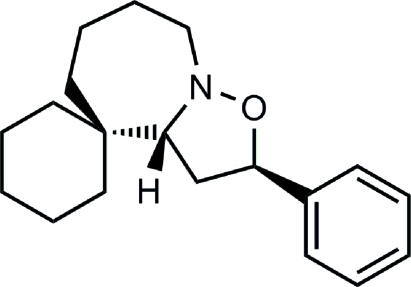


## Experimental

### 

#### Crystal data

C_19_H_27_NO
*M* *_r_* = 285.42
Triclinic, 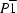

*a* = 9.8516 (1) Å
*b* = 10.4560 (1) Å
*c* = 16.0957 (1) Å
α = 101.058 (1)°
β = 92.833 (1)°
γ = 96.527 (1)°
*V* = 1612.34 (2) Å^3^
*Z* = 4
Mo *K*α radiation
μ = 0.07 mm^−1^
*T* = 85 (2) K
0.32 × 0.24 × 0.22 mm

#### Data collection

Siemens SMART CCD diffractometer
Absorption correction: multi-scan (*SADABS*; Sheldrick, 1996 ▶) *T*_min_ = 0.978, *T*_max_ = 0.985
15138 measured reflections
6437 independent reflections
4959 reflections with *I* > 2σ(*I*)
*R*_int_ = 0.026

#### Refinement

*R*[*F* ^2^ > 2σ(*F* ^2^)] = 0.084
*wR*(*F* ^2^) = 0.194
*S* = 1.06
6437 reflections
379 parameters
H-atom parameters constrained
Δρ_max_ = 1.21 e Å^−3^
Δρ_min_ = −0.32 e Å^−3^

### 

Data collection: *SMART* (Siemens, 1995 ▶); cell refinement: *SAINT* (Siemens, 1995 ▶); data reduction: *SAINT*; program(s) used to solve structure: *SHELXS97* (Sheldrick, 2008 ▶); program(s) used to refine structure: *SHELXL97* (Sheldrick, 2008 ▶); molecular graphics: *ORTEPIII* (Burnett & Johnson, 1996 ▶) and *Mercury* (Macrae *et al.*, 2006 ▶); software used to prepare material for publication: *SHELXTL* (Sheldrick, 2008 ▶) and *publCIF* (Westrip, 2008 ▶).

## Supplementary Material

Crystal structure: contains datablocks I, global. DOI: 10.1107/S1600536808021867/bi2294sup1.cif

Structure factors: contains datablocks I. DOI: 10.1107/S1600536808021867/bi2294Isup2.hkl

Additional supplementary materials:  crystallographic information; 3D view; checkCIF report

## Figures and Tables

**Fig. 1.**
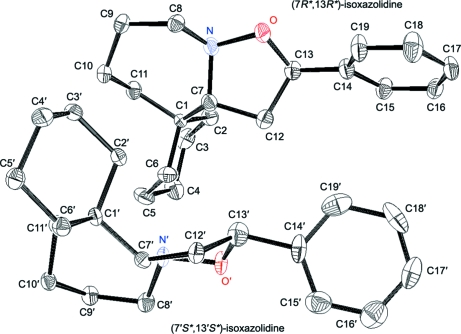
The molecular structure and atom numbering scheme of (7R*,13R* and 7S*,13S*)-isoxazolidine with displacement ellipsoids drawn at the 50% probability level. H atoms are omitted.

**Fig. 2.**
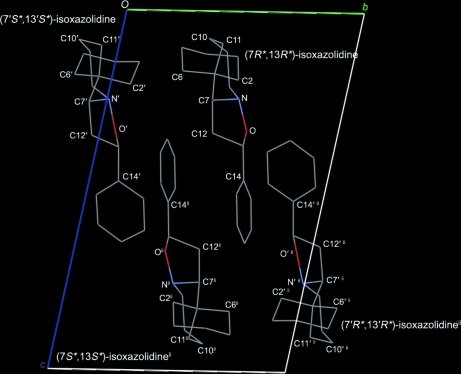
Molecular packing of racemic isoxazolidine. H atoms are omitted. The origin of the unit cell is labelled as O while cell axes are labelled as a (red), b (green) and c (blue), respectively. [Symmetry code: (ii) 1 - x, 1 - y, 1 - z.]

## supplementary crystallographic information

### Comment

The title isoxazolidine was prepared as part of a synthetic program (Brimble & Trzoss, 2004; Brimble *et al.* 2005; O'Connor *et al.*, 2008 and Guéret *et al.*, 2008) directed towards the synthesis of heterocycles related to the spiroimine unit of the spirolide family of shellfish toxins that were isolated from the culture of a toxic clone of the dinoflagellate *Alexandrium ostenfeldii* (Hu *et al.*, 2001; MacKinnon *et al.*, 2006 and Ciminiello *et al.*, 2007). With this idea in mind, a study of the thermal-promoted 1,3-dipolar cycloaddition of a novel 7,6-spironitrone to a range of alkenes was investigated. In particular, the addition of the 7,6-spironitrone to styrene proceeded regioselectively to afford the title racemic isoxazolidine. However, establishment of the *exo* or *endo* selectivity of this 1,3-dipolar cycloaddition was required and this could not be deduced from detailed NMR studies. The crystal structure of the cycloadduct (Fig. 1) clearly indicates the *syn* stereochemistry between the phenyl group at C13 and the bridgehead proton at C7, thereby confirming that the 1,3-dipolar cycloaddition took place with *exo* selectivity.

The relative stereochemistry of the racemic isoxazolidine is shown to be 7*R**,13*R** and 7*S**,13*S** from the crystal structure. Apart from the opposite stereochemistry, the two molecules in the asymmetric unit also differ from each other in the orientation adopted by the phenyl ring relative to the adjacent isoxazolidine ring. This is shown by the different torsion angle adopted at C12–C13–C14–C15: for one enantiomer, the torsion angle is 63.6 (4)° while the torsion angle for the opposite enantiomer is 86.8 (4)°. In both molecules, the six-membered ring adopts a chair conformation while the seven-membered ring adopts a twist-chair conformation. A view of the molecular packing is depicted in Fig. 2.

### Experimental

8-Azoniaspiro[5.6]dodec-7-en-8-olate (550 mg, 1.96 mmol) and styrene (905 µl, 7.9 mmol) were dissolved in toluene (10 ml) under an argon atmosphere and the solution was heated under reflux for 12 h. The reaction mixture was cooled to room temperature and the solvent was concentrated *in vacuo*. The residue was purified directly by flash chromatography (hexane-ethyl acetate 10:1) to give the title compound as a white solid (353 mg, 63%). Recrystallization from diethyl ether afforded white needles.

### Refinement

H atoms were placed in calculated positions and were refined using a riding model (C–H = 0.93 or 0.97 Å), with *U*_iso_(H) = 1.2 or 1.5 times *U*_eq_(C). Two peaks are present in the final difference density: 1.21 eÅ^-3^ at a distance of 1.11 Å from O, and 1.02 eÅ^-3^ at a distance of 1.20 Å from C14.

### Crystal data

**Table:**

C_19_H_27_NO	*Z* = 4
*M**_r_* = 285.42	*F*_000_ = 624
Triclinic, *P*1	*D*_x_ = 1.176 Mg m^−^^3^
Hall symbol: -P 1	Melting point: 338.8(7) K
*a* = 9.8516 (1) Å	Mo *K*α radiation λ = 0.71073 Å
*b* = 10.4560 (1) Å	Cell parameters from 6437 reflections
*c* = 16.0957 (1) Å	θ = 1.3–26.4º
α = 101.058 (1)º	µ = 0.07 mm^−^^1^
β = 92.833 (1)º	*T* = 85 (2) K
γ = 96.527 (1)º	Needles, white
*V* = 1612.34 (2) Å^3^	0.32 × 0.24 × 0.22 mm

### Data collection

**Table:**

Siemens SMART CCD diffractometer	6437 independent reflections
Radiation source: fine-focus sealed tube	4959 reflections with *I* > 2σ(*I*)
Monochromator: graphite	*R*_int_ = 0.026
*T* = 85(2) K	θ_max_ = 26.4º
ω scans	θ_min_ = 1.3º
Absorption correction: multi-scan(SADABS; Sheldrick, 1996)	*h* = −12→12
*T*_min_ = 0.978, *T*_max_ = 0.985	*k* = −13→12
15138 measured reflections	*l* = 0→20

### Refinement

**Table:**

Refinement on *F*^2^	Secondary atom site location: difference Fourier map
Least-squares matrix: full	Hydrogen site location: inferred from neighbouring sites
*R*[*F*^2^ > 2σ(*F*^2^)] = 0.084	H-atom parameters constrained
*wR*(*F*^2^) = 0.194	*w* = 1/[σ^2^(*F*_o_^2^) + (0.0475*P*)^2^ + 4.1372*P*] where *P* = (*F*_o_^2^ + 2*F*_c_^2^)/3
*S* = 1.06	(Δ/σ)_max_ < 0.001
6437 reflections	Δρ_max_ = 1.21 e Å^−^^3^
379 parameters	Δρ_min_ = −0.32 e Å^−^^3^
Primary atom site location: structure-invariant direct methods	Extinction correction: none

### Special details

**Table:**

Geometry. All e.s.d.'s (except the e.s.d. in the dihedral angle between two l.s. planes) are estimated using the full covariance matrix. The cell e.s.d.'s are taken into account individually in the estimation of e.s.d.'s in distances, angles and torsion angles; correlations between e.s.d.'s in cell parameters are only used when they are defined by crystal symmetry. An approximate (isotropic) treatment of cell e.s.d.'s is used for estimating e.s.d.'s involving l.s. planes.
Refinement. Refinement of *F*^2^ against ALL reflections. The weighted *R*-factor *wR* and goodness of fit *S* are based on *F*^2^, conventional *R*-factors *R* are based on *F*, with *F* set to zero for negative *F*^2^. The threshold expression of *F*^2^ > 2σ(*F*^2^) is used only for calculating *R*-factors(gt) *etc*. and is not relevant to the choice of reflections for refinement. *R*-factors based on *F*^2^ are statistically about twice as large as those based on *F*, and *R*- factors based on ALL data will be even larger.

### Fractional atomic coordinates and isotropic or equivalent isotropic displacement parameters (Å^2^)

**Table:**

	*x*	*y*	*z*	*U*_iso_*/*U*_eq_	
O	0.6455 (2)	0.6154 (2)	0.33169 (13)	0.0268 (5)	
N	0.6590 (3)	0.5526 (3)	0.24237 (15)	0.0226 (6)	
C1	0.8405 (3)	0.4282 (3)	0.17312 (17)	0.0161 (6)	
C2	0.9653 (3)	0.5340 (3)	0.1897 (2)	0.0243 (7)	
H2A	0.9346	0.6189	0.1894	0.029*	
H2B	1.0085	0.5368	0.2456	0.029*	
C3	1.0710 (3)	0.5099 (3)	0.1238 (2)	0.0303 (7)	
H3A	1.0313	0.5166	0.0687	0.036*	
H3B	1.1496	0.5768	0.1390	0.036*	
C4	1.1179 (3)	0.3742 (3)	0.1186 (2)	0.0347 (8)	
H4A	1.1678	0.3714	0.1715	0.042*	
H4B	1.1796	0.3592	0.0735	0.042*	
C5	0.9964 (3)	0.2656 (3)	0.1013 (2)	0.0319 (8)	
H5A	1.0288	0.1820	0.1033	0.038*	
H5B	0.9542	0.2603	0.0448	0.038*	
C6	0.8892 (3)	0.2914 (3)	0.1667 (2)	0.0252 (7)	
H6A	0.9280	0.2842	0.2219	0.030*	
H6B	0.8107	0.2245	0.1511	0.030*	
C7	0.7425 (3)	0.4433 (3)	0.24678 (18)	0.0219 (6)	
H7A	0.6798	0.3616	0.2397	0.026*	
C8	0.5176 (3)	0.5018 (3)	0.20965 (18)	0.0213 (6)	
H8A	0.4585	0.5690	0.2264	0.026*	
H8B	0.4865	0.4267	0.2341	0.026*	
C9	0.5073 (3)	0.4610 (3)	0.11317 (18)	0.0229 (6)	
H9A	0.4157	0.4169	0.0949	0.028*	
H9B	0.5186	0.5399	0.0896	0.028*	
C10	0.6094 (3)	0.3717 (3)	0.07546 (19)	0.0238 (7)	
H10A	0.5858	0.3437	0.0150	0.029*	
H10B	0.6010	0.2939	0.1003	0.029*	
C11	0.7593 (3)	0.4353 (3)	0.08922 (18)	0.0228 (6)	
H11A	0.7609	0.5272	0.0861	0.027*	
H11B	0.8080	0.3946	0.0423	0.027*	
C12	0.8092 (3)	0.4744 (3)	0.33864 (19)	0.0249 (7)	
H12A	0.7685	0.4139	0.3719	0.030*	
H12B	0.9071	0.4705	0.3393	0.030*	
C13	0.7780 (3)	0.6141 (3)	0.37195 (19)	0.0239 (7)	
H13A	0.8452	0.6775	0.3535	0.029*	
C14	0.7717 (3)	0.6482 (3)	0.46735 (19)	0.0254 (7)	
C15	0.8925 (3)	0.6960 (3)	0.51785 (19)	0.0275 (7)	
H15A	0.9748	0.7075	0.4926	0.033*	
C16	0.8910 (3)	0.7266 (3)	0.60540 (19)	0.0271 (7)	
H16A	0.9722	0.7578	0.6387	0.033*	
C17	0.7693 (4)	0.7109 (3)	0.6432 (2)	0.0324 (8)	
H17A	0.7683	0.7323	0.7020	0.039*	
C18	0.6486 (4)	0.6633 (4)	0.5938 (2)	0.0414 (9)	
H18A	0.5666	0.6529	0.6194	0.050*	
C19	0.6496 (3)	0.6311 (4)	0.5058 (2)	0.0333 (8)	
H19A	0.5685	0.5981	0.4728	0.040*	
O'	0.8141 (2)	0.0639 (2)	0.33601 (12)	0.0231 (5)	
N'	0.7585 (2)	0.0078 (2)	0.24822 (14)	0.0186 (5)	
C1'	0.5197 (3)	−0.0548 (3)	0.18622 (17)	0.0159 (6)	
C2'	0.4734 (3)	0.0835 (3)	0.20648 (17)	0.0172 (6)	
H2'A	0.4426	0.0989	0.2634	0.021*	
H2'B	0.5512	0.1487	0.2051	0.021*	
C3'	0.3578 (3)	0.1009 (3)	0.14386 (18)	0.0210 (6)	
H3'A	0.3322	0.1888	0.1592	0.025*	
H3'B	0.3892	0.0898	0.0870	0.025*	
C4'	0.2331 (3)	−0.0004 (3)	0.1454 (2)	0.0250 (7)	
H4'A	0.1980	0.0148	0.2012	0.030*	
H4'B	0.1614	0.0096	0.1045	0.030*	
C5'	0.2713 (3)	−0.1402 (3)	0.1242 (2)	0.0234 (6)	
H5'A	0.2940	−0.1593	0.0655	0.028*	
H5'B	0.1930	−0.2019	0.1304	0.028*	
C6'	0.3936 (3)	−0.1592 (3)	0.18186 (19)	0.0190 (6)	
H6'A	0.4205	−0.2454	0.1615	0.023*	
H6'B	0.3644	−0.1569	0.2387	0.023*	
C7'	0.6255 (3)	−0.0735 (3)	0.25610 (17)	0.0161 (6)	
H7'A	0.6404	−0.1661	0.2461	0.019*	
C8'	0.8619 (3)	−0.0742 (3)	0.21325 (19)	0.0227 (6)	
H8'A	0.8582	−0.1516	0.2384	0.027*	
H8'B	0.9525	−0.0253	0.2272	0.027*	
C9'	0.8360 (3)	−0.1158 (3)	0.11659 (18)	0.0221 (6)	
H9'A	0.8963	−0.1805	0.0965	0.027*	
H9'B	0.8611	−0.0398	0.0916	0.027*	
C10'	0.6885 (3)	−0.1737 (3)	0.08409 (18)	0.0196 (6)	
H10C	0.6601	−0.2454	0.1122	0.024*	
H10D	0.6863	−0.2095	0.0237	0.024*	
C11'	0.5856 (3)	−0.0725 (3)	0.09918 (17)	0.0181 (6)	
H11C	0.5124	−0.0976	0.0544	0.022*	
H11D	0.6319	0.0121	0.0936	0.022*	
C12'	0.5939 (3)	−0.0310 (3)	0.34989 (18)	0.0207 (6)	
H12C	0.5004	−0.0109	0.3543	0.025*	
H12D	0.6083	−0.0987	0.3818	0.025*	
C13'	0.6976 (4)	0.0927 (4)	0.3809 (2)	0.0321 (8)	
H13B	0.6620	0.1689	0.3646	0.039*	
C14'	0.7431 (3)	0.1240 (3)	0.47534 (19)	0.0287 (7)	
C15'	0.8144 (4)	0.0435 (4)	0.5145 (2)	0.0370 (8)	
H15B	0.8305	−0.0371	0.4832	0.044*	
C16'	0.8617 (4)	0.0779 (4)	0.5967 (3)	0.0420 (9)	
H16B	0.9115	0.0222	0.6209	0.050*	
C17'	0.8369 (4)	0.1928 (4)	0.6440 (2)	0.0367 (9)	
H17B	0.8691	0.2142	0.7009	0.044*	
C18'	0.7657 (4)	0.2789 (4)	0.6110 (2)	0.0399 (9)	
H18B	0.7493	0.3575	0.6449	0.048*	
C19'	0.7168 (4)	0.2452 (4)	0.5227 (2)	0.0374 (9)	
H19B	0.6693	0.3018	0.4979	0.045*	

### Atomic displacement parameters (Å^2^)

**Table:**

	*U*^11^	*U*^22^	*U*^33^	*U*^12^	*U*^13^	*U*^23^
O	0.0293 (12)	0.0325 (12)	0.0177 (11)	0.0086 (10)	0.0025 (9)	−0.0005 (9)
N	0.0224 (13)	0.0262 (14)	0.0178 (12)	0.0041 (10)	0.0022 (10)	0.0001 (10)
C1	0.0192 (14)	0.0138 (13)	0.0145 (13)	0.0037 (11)	0.0001 (11)	0.0005 (10)
C2	0.0219 (15)	0.0196 (15)	0.0301 (17)	−0.0010 (12)	−0.0013 (13)	0.0046 (12)
C3	0.0240 (16)	0.0380 (19)	0.0284 (17)	−0.0063 (14)	−0.0009 (13)	0.0120 (14)
C4	0.0202 (16)	0.042 (2)	0.0372 (19)	0.0034 (14)	0.0091 (14)	−0.0053 (16)
C5	0.0310 (18)	0.0240 (17)	0.040 (2)	0.0096 (14)	0.0065 (15)	0.0000 (14)
C6	0.0241 (16)	0.0220 (16)	0.0305 (17)	0.0053 (12)	0.0016 (13)	0.0066 (13)
C7	0.0270 (16)	0.0177 (14)	0.0201 (15)	0.0011 (12)	0.0006 (12)	0.0024 (11)
C8	0.0174 (14)	0.0245 (15)	0.0220 (15)	0.0021 (12)	0.0010 (12)	0.0048 (12)
C9	0.0191 (15)	0.0273 (16)	0.0207 (15)	−0.0011 (12)	−0.0024 (12)	0.0041 (12)
C10	0.0263 (16)	0.0249 (16)	0.0183 (15)	0.0028 (13)	−0.0030 (12)	0.0007 (12)
C11	0.0240 (16)	0.0260 (16)	0.0176 (14)	0.0020 (12)	0.0030 (12)	0.0024 (12)
C12	0.0262 (16)	0.0267 (16)	0.0215 (15)	0.0017 (13)	−0.0001 (12)	0.0057 (12)
C13	0.0226 (15)	0.0272 (16)	0.0211 (15)	0.0014 (13)	0.0016 (12)	0.0037 (12)
C14	0.0248 (16)	0.0300 (17)	0.0189 (15)	0.0024 (13)	0.0021 (12)	−0.0007 (13)
C15	0.0239 (16)	0.0377 (19)	0.0204 (15)	0.0019 (14)	0.0027 (12)	0.0052 (13)
C16	0.0330 (18)	0.0259 (16)	0.0204 (15)	0.0001 (13)	−0.0059 (13)	0.0034 (12)
C17	0.043 (2)	0.0352 (19)	0.0162 (15)	0.0062 (15)	0.0028 (14)	−0.0023 (13)
C18	0.0274 (18)	0.068 (3)	0.0247 (18)	0.0012 (17)	0.0085 (14)	0.0000 (17)
C19	0.0243 (17)	0.050 (2)	0.0194 (16)	−0.0016 (15)	0.0021 (13)	−0.0039 (15)
O'	0.0212 (11)	0.0300 (12)	0.0135 (10)	−0.0037 (9)	−0.0021 (8)	−0.0023 (8)
N'	0.0180 (12)	0.0218 (13)	0.0128 (11)	−0.0035 (10)	−0.0024 (9)	0.0001 (9)
C1'	0.0177 (14)	0.0144 (13)	0.0136 (13)	−0.0011 (11)	0.0004 (11)	0.0002 (10)
C2'	0.0204 (14)	0.0150 (14)	0.0152 (13)	0.0010 (11)	0.0024 (11)	0.0013 (10)
C3'	0.0253 (15)	0.0190 (15)	0.0193 (14)	0.0073 (12)	0.0020 (12)	0.0027 (11)
C4'	0.0186 (15)	0.0265 (16)	0.0295 (17)	0.0065 (12)	−0.0008 (12)	0.0030 (13)
C5'	0.0167 (14)	0.0229 (16)	0.0276 (16)	−0.0021 (12)	−0.0034 (12)	0.0018 (12)
C6'	0.0171 (14)	0.0162 (14)	0.0227 (15)	−0.0008 (11)	0.0004 (11)	0.0032 (11)
C7'	0.0164 (14)	0.0135 (13)	0.0179 (14)	−0.0001 (10)	0.0021 (11)	0.0030 (10)
C8'	0.0166 (14)	0.0283 (16)	0.0217 (15)	0.0027 (12)	0.0007 (12)	0.0018 (12)
C9'	0.0187 (15)	0.0252 (16)	0.0213 (15)	0.0013 (12)	0.0026 (12)	0.0021 (12)
C10'	0.0199 (14)	0.0213 (15)	0.0160 (14)	0.0035 (12)	0.0017 (11)	−0.0009 (11)
C11'	0.0174 (14)	0.0209 (15)	0.0149 (13)	0.0030 (11)	0.0007 (11)	0.0009 (11)
C12'	0.0222 (15)	0.0231 (15)	0.0154 (14)	0.0000 (12)	0.0004 (11)	0.0026 (11)
C13'	0.0323 (18)	0.0367 (19)	0.0263 (17)	0.0035 (15)	0.0017 (14)	0.0043 (14)
C14'	0.0202 (15)	0.044 (2)	0.0161 (15)	−0.0059 (14)	−0.0006 (12)	−0.0018 (13)
C15'	0.035 (2)	0.0290 (18)	0.047 (2)	0.0034 (15)	−0.0019 (16)	0.0073 (16)
C16'	0.041 (2)	0.043 (2)	0.043 (2)	−0.0023 (17)	−0.0005 (17)	0.0170 (18)
C17'	0.0293 (18)	0.058 (2)	0.0209 (17)	−0.0085 (17)	−0.0013 (14)	0.0122 (16)
C18'	0.035 (2)	0.0308 (19)	0.045 (2)	−0.0044 (15)	0.0185 (17)	−0.0152 (16)
C19'	0.0246 (17)	0.045 (2)	0.050 (2)	0.0079 (15)	0.0074 (16)	0.0244 (18)

### Geometric parameters (Å, °)

**Table:**

O—C13	1.431 (4)	O'—C13'	1.417 (4)
O—N	1.480 (3)	O'—N'	1.474 (3)
N—C8	1.466 (4)	N'—C8'	1.469 (4)
N—C7	1.493 (4)	N'—C7'	1.503 (3)
C1—C2	1.533 (4)	C1'—C2'	1.546 (4)
C1—C6	1.545 (4)	C1'—C6'	1.547 (4)
C1—C11	1.554 (4)	C1'—C7'	1.551 (4)
C1—C7	1.561 (4)	C1'—C11'	1.561 (4)
C2—C3	1.529 (4)	C2'—C3'	1.533 (4)
C2—H2A	0.970	C2'—H2'A	0.970
C2—H2B	0.970	C2'—H2'B	0.970
C3—C4	1.529 (5)	C3'—C4'	1.532 (4)
C3—H3A	0.970	C3'—H3'A	0.970
C3—H3B	0.970	C3'—H3'B	0.970
C4—C5	1.529 (5)	C4'—C5'	1.530 (4)
C4—H4A	0.970	C4'—H4'A	0.970
C4—H4B	0.970	C4'—H4'B	0.970
C5—C6	1.537 (4)	C5'—C6'	1.538 (4)
C5—H5A	0.970	C5'—H5'A	0.970
C5—H5B	0.970	C5'—H5'B	0.970
C6—H6A	0.970	C6'—H6'A	0.970
C6—H6B	0.970	C6'—H6'B	0.970
C7—C12	1.547 (4)	C7'—C12'	1.547 (4)
C7—H7A	0.980	C7'—H7'A	0.980
C8—C9	1.525 (4)	C8'—C9'	1.533 (4)
C8—H8A	0.970	C8'—H8'A	0.970
C8—H8B	0.970	C8'—H8'B	0.970
C9—C10	1.519 (4)	C9'—C10'	1.535 (4)
C9—H9A	0.970	C9'—H9'A	0.970
C9—H9B	0.970	C9'—H9'B	0.970
C10—C11	1.536 (4)	C10'—C11'	1.542 (4)
C10—H10A	0.970	C10'—H10C	0.970
C10—H10B	0.970	C10'—H10D	0.970
C11—H11A	0.970	C11'—H11C	0.970
C11—H11B	0.970	C11'—H11D	0.970
C12—C13	1.527 (4)	C12'—C13'	1.539 (4)
C12—H12A	0.970	C12'—H12C	0.970
C12—H12B	0.970	C12'—H12D	0.970
C13—C14	1.514 (4)	C13'—C14'	1.525 (4)
C13—H13A	0.980	C13'—H13B	0.980
C14—C19	1.390 (4)	C14'—C15'	1.376 (5)
C14—C15	1.393 (4)	C14'—C19'	1.407 (5)
C15—C16	1.385 (4)	C15'—C16'	1.348 (5)
C15—H15A	0.930	C15'—H15B	0.930
C16—C17	1.379 (5)	C16'—C17'	1.348 (5)
C16—H16A	0.930	C16'—H16B	0.930
C17—C18	1.383 (5)	C17'—C18'	1.370 (6)
C17—H17A	0.930	C17'—H17B	0.930
C18—C19	1.392 (5)	C18'—C19'	1.441 (5)
C18—H18A	0.930	C18'—H18B	0.930
C19—H19A	0.930	C19'—H19B	0.930
			
C13—O—N	103.2 (2)	C13'—O'—N'	104.6 (2)
C8—N—O	103.8 (2)	C8'—N'—O'	103.8 (2)
C8—N—C7	111.2 (2)	C8'—N'—C7'	111.8 (2)
O—N—C7	105.4 (2)	O'—N'—C7'	105.51 (19)
C2—C1—C6	109.4 (2)	C2'—C1'—C6'	109.1 (2)
C2—C1—C11	109.5 (2)	C2'—C1'—C7'	111.1 (2)
C6—C1—C11	110.1 (2)	C6'—C1'—C7'	108.3 (2)
C2—C1—C7	112.5 (2)	C2'—C1'—C11'	108.8 (2)
C6—C1—C7	106.4 (2)	C6'—C1'—C11'	110.1 (2)
C11—C1—C7	108.9 (2)	C7'—C1'—C11'	109.4 (2)
C3—C2—C1	112.9 (3)	C3'—C2'—C1'	112.7 (2)
C3—C2—H2A	109.0	C3'—C2'—H2'A	109.1
C1—C2—H2A	109.0	C1'—C2'—H2'A	109.1
C3—C2—H2B	109.0	C3'—C2'—H2'B	109.1
C1—C2—H2B	109.0	C1'—C2'—H2'B	109.1
H2A—C2—H2B	107.8	H2'A—C2'—H2'B	107.8
C4—C3—C2	111.2 (3)	C4'—C3'—C2'	110.0 (2)
C4—C3—H3A	109.4	C4'—C3'—H3'A	109.7
C2—C3—H3A	109.4	C2'—C3'—H3'A	109.7
C4—C3—H3B	109.4	C4'—C3'—H3'B	109.7
C2—C3—H3B	109.4	C2'—C3'—H3'B	109.7
H3A—C3—H3B	108.0	H3'A—C3'—H3'B	108.2
C3—C4—C5	111.5 (3)	C5'—C4'—C3'	110.9 (2)
C3—C4—H4A	109.3	C5'—C4'—H4'A	109.5
C5—C4—H4A	109.3	C3'—C4'—H4'A	109.5
C3—C4—H4B	109.3	C5'—C4'—H4'B	109.5
C5—C4—H4B	109.3	C3'—C4'—H4'B	109.5
H4A—C4—H4B	108.0	H4'A—C4'—H4'B	108.1
C4—C5—C6	111.4 (3)	C4'—C5'—C6'	112.1 (2)
C4—C5—H5A	109.3	C4'—C5'—H5'A	109.2
C6—C5—H5A	109.3	C6'—C5'—H5'A	109.2
C4—C5—H5B	109.3	C4'—C5'—H5'B	109.2
C6—C5—H5B	109.3	C6'—C5'—H5'B	109.2
H5A—C5—H5B	108.0	H5'A—C5'—H5'B	107.9
C5—C6—C1	112.9 (3)	C5'—C6'—C1'	114.1 (2)
C5—C6—H6A	109.0	C5'—C6'—H6'A	108.7
C1—C6—H6A	109.0	C1'—C6'—H6'A	108.7
C5—C6—H6B	109.0	C5'—C6'—H6'B	108.7
C1—C6—H6B	109.0	C1'—C6'—H6'B	108.7
H6A—C6—H6B	107.8	H6'A—C6'—H6'B	107.6
N—C7—C12	104.7 (2)	N'—C7'—C12'	104.8 (2)
N—C7—C1	110.6 (2)	N'—C7'—C1'	108.3 (2)
C12—C7—C1	117.3 (2)	C12'—C7'—C1'	118.0 (2)
N—C7—H7A	108.0	N'—C7'—H7'A	108.5
C12—C7—H7A	108.0	C12'—C7'—H7'A	108.5
C1—C7—H7A	108.0	C1'—C7'—H7'A	108.5
N—C8—C9	111.0 (2)	N'—C8'—C9'	110.4 (2)
N—C8—H8A	109.4	N'—C8'—H8'A	109.6
C9—C8—H8A	109.4	C9'—C8'—H8'A	109.6
N—C8—H8B	109.4	N'—C8'—H8'B	109.6
C9—C8—H8B	109.4	C9'—C8'—H8'B	109.6
H8A—C8—H8B	108.0	H8'A—C8'—H8'B	108.1
C10—C9—C8	116.2 (3)	C8'—C9'—C10'	115.6 (2)
C10—C9—H9A	108.2	C8'—C9'—H9'A	108.4
C8—C9—H9A	108.2	C10'—C9'—H9'A	108.4
C10—C9—H9B	108.2	C8'—C9'—H9'B	108.4
C8—C9—H9B	108.2	C10'—C9'—H9'B	108.4
H9A—C9—H9B	107.4	H9'A—C9'—H9'B	107.5
C9—C10—C11	114.3 (2)	C9'—C10'—C11'	113.3 (2)
C9—C10—H10A	108.7	C9'—C10'—H10C	108.9
C11—C10—H10A	108.7	C11'—C10'—H10C	108.9
C9—C10—H10B	108.7	C9'—C10'—H10D	108.9
C11—C10—H10B	108.7	C11'—C10'—H10D	108.9
H10A—C10—H10B	107.6	H10C—C10'—H10D	107.7
C10—C11—C1	117.6 (2)	C10'—C11'—C1'	116.6 (2)
C10—C11—H11A	107.9	C10'—C11'—H11C	108.1
C1—C11—H11A	107.9	C1'—C11'—H11C	108.1
C10—C11—H11B	107.9	C10'—C11'—H11D	108.1
C1—C11—H11B	107.9	C1'—C11'—H11D	108.1
H11A—C11—H11B	107.2	H11C—C11'—H11D	107.3
C13—C12—C7	102.9 (2)	C13'—C12'—C7'	102.6 (2)
C13—C12—H12A	111.2	C13'—C12'—H12C	111.3
C7—C12—H12A	111.2	C7'—C12'—H12C	111.3
C13—C12—H12B	111.2	C13'—C12'—H12D	111.3
C7—C12—H12B	111.2	C7'—C12'—H12D	111.3
H12A—C12—H12B	109.1	H12C—C12'—H12D	109.2
O—C13—C14	109.4 (2)	O'—C13'—C14'	107.4 (3)
O—C13—C12	103.1 (2)	O'—C13'—C12'	103.4 (3)
C14—C13—C12	114.1 (3)	C14'—C13'—C12'	116.3 (3)
O—C13—H13A	110.0	O'—C13'—H13B	109.8
C14—C13—H13A	110.0	C14'—C13'—H13B	109.8
C12—C13—H13A	110.0	C12'—C13'—H13B	109.8
C19—C14—C15	119.2 (3)	C15'—C14'—C19'	119.1 (3)
C19—C14—C13	121.9 (3)	C15'—C14'—C13'	123.3 (3)
C15—C14—C13	118.8 (3)	C19'—C14'—C13'	117.4 (3)
C16—C15—C14	120.4 (3)	C16'—C15'—C14'	122.0 (4)
C16—C15—H15A	119.8	C16'—C15'—H15B	119.0
C14—C15—H15A	119.8	C14'—C15'—H15B	119.0
C17—C16—C15	120.1 (3)	C17'—C16'—C15'	120.2 (4)
C17—C16—H16A	119.9	C17'—C16'—H16B	119.9
C15—C16—H16A	119.9	C15'—C16'—H16B	119.9
C16—C17—C18	120.0 (3)	C16'—C17'—C18'	122.1 (3)
C16—C17—H17A	120.0	C16'—C17'—H17B	118.9
C18—C17—H17A	120.0	C18'—C17'—H17B	118.9
C17—C18—C19	120.2 (3)	C17'—C18'—C19'	118.6 (3)
C17—C18—H18A	119.9	C17'—C18'—H18B	120.7
C19—C18—H18A	119.9	C19'—C18'—H18B	120.7
C14—C19—C18	120.0 (3)	C14'—C19'—C18'	117.9 (3)
C14—C19—H19A	120.0	C14'—C19'—H19B	121.0
C18—C19—H19A	120.0	C18'—C19'—H19B	121.0
